# DNA polymerase gamma variants and hepatotoxicity during maintenance therapy of childhood acute lymphoblastic leukemia: is there a causal relationship?

**DOI:** 10.1038/s41397-023-00303-0

**Published:** 2023-05-03

**Authors:** Tekla Harju, Anri Hurme-Niiranen, Maria Suo-Palosaari, Stine Nygaard Nielsen, Reetta Hinttala, Kjeld Schmiegelow, Johanna Uusimaa, Arja Harila, Riitta Niinimäki

**Affiliations:** 1 Research Unit of Clinical Medicine, University of Oulu, Oulu, Finland; 2 Department of Children and Adolescents, Oulu University Hospital, Oulu, Finland; 3 Medical Research Center, University of Oulu and Oulu University Hospital, Oulu, Finland; 4 Department of Diagnostic Radiology, Oulu University Hospital, Oulu, Finland; 5 Research Unit of Health Sciences and Technology, Oulu University Hospital and University of Oulu, Oulu, Finland; 6 Department of Pediatrics and Adolescent Medicine, Rigshospitalet, Copenhagen University Hospital, and Institute of Clinical Medicine, University of Copenhagen, Copenhagen, Denmark; 7 Biocenter Oulu, University of Oulu, Oulu, Finland; 8 Pediatric Oncology Laboratory, Rigshospitalet, Copenhagen University Hospital, Copenhagen, Denmark; 9 Department of Women’s and Children’s Health, Uppsala University, Uppsala, Sweden

**Keywords:** Medical genetics, Translational research, Cancer genetics, Haematological diseases

## Abstract

Hepatotoxicity is a frequent complication during maintenance therapy of acute lymphoblastic leukemia (ALL) with 6-mercaptopurine and methotrexate. Elevated levels of methylated 6-mercaptopurine metabolites (MeMP) are associated with hepatotoxicity. However, not all mechanisms are known that lead to liver failure in patients with ALL. Variants in the *POLG* gene, which encodes the catalytic subunit of mitochondrial DNA polymerase gamma (POLG1), have been related to drug-induced hepatotoxicity, for example, by sodium valproate. The association of common *POLG* variants with hepatotoxicity during maintenance therapy was studied in 34 patients with childhood ALL. Of the screened *POLG* variants, four different variants were detected in 12 patients. One patient developed severe hepatotoxicity without elevated MeMP levels and harbored a heterozygous POLG p.G517V variant, which was not found in the other patients.

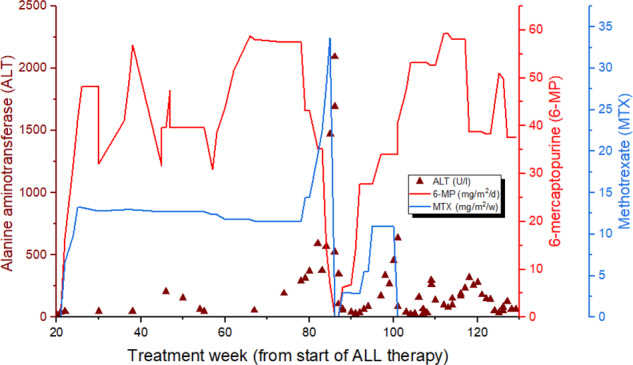

## Introduction

Treatment-related hepatotoxicity, ranging from mild symptoms to severe veno-occlusive disease, is common in acute lymphoblastic leukemia (ALL) [1, 2]. Hepatotoxicity during maintenance is related to the oral maintenance therapy drugs 6-mercaptopurine (6-MP) and methotrexate (MTX) and is usually associated with increased methylated 6-mercaptopurine metabolite (MeMP) levels and the wild-type thiopurine methyltransferase (*TPMT*) genotype involved in 6-MP metabolism [3, 4]. Still, maintenance therapy–associated hepatotoxicity shows wide inter-individual variation and is suggested to be associated with other genetic risk factors, especially in patients who do not show elevated MeMP levels.

Human DNA polymerase gamma (Pol γ) replicates, maintains, and repairs mitochondrial DNA (mtDNA), and it is a heterotrimer formed by the catalytic domain POLG1, encoded by *POLG* at chromosomal locus 15q25, and two accessory subunits of POLG2 [5]. Drug-induced damage to the mitochondrial genome, either by direct impact on the DNA or by impaired Pol γ function, leads to aberrant mitochondrial protein synthesis and respiratory chain function, resulting in oxidative stress, altered mitochondrial membrane properties, and impaired metabolic functions [6]. In hepatocytes, this mitochondrial dysfunction contributes to cell death and triglyceride accumulation (i.e., steatosis) and, thus, drug-induced liver injury [7]. The association of Pol γ deficiency with liver dysfunction was first described in patients with Alpers’ disease [8]. Since then, several studies have demonstrated that patients with homozygous or compound heterozygous pathogenic variants on POLG1 (e.g., p.W748S and p.A467T) are at greater risk of liver failure induced by sodium valproate (VPA) [9, 10].

In this study, we investigated the association of chemotherapy-related hepatotoxicity during maintenance with common variants of the catalytic POLG1 domain of Pol γ in 34 patients diagnosed with childhood ALL.

## Patients and methods

### Patient cohort

Nine common POLG1 variants (p.T251I, p.A467T, p.N468D, p.G517V, p.P587L, p.R722H, p.W748S, p.E1143G, and p.Q1236H) were analyzed in 34 Finnish patients (18 boys and 16 girls; median age at diagnosis: 5 years [range: 2–15 years]) who were diagnosed consecutively between 2008 and 2013 in a tertiary level hospital. Exclusion criteria were Down syndrome, stem cell transplantation, or infant leukemia. All patients were treated according to the Nordic Society of Pediatric Hematology and Oncology (NOPHO) ALL2008 treatment protocol, 16 according to the standard risk (SR) protocol, 15 according to the intermediate risk (IR) protocol [1], and three according to the high risk (HR) protocol [11].

Medical records of the patients were reviewed for clinical signs of hepatotoxicity during maintenance therapy. The highest serum alanine aminotransferase (ALT) and bilirubin levels were defined after the start of maintenance therapy at week 20 in SR, week 22 in IR, and week 36 in the HR treatment protocol. The levels were assessed before or at least two weeks after the administration of high-dose MTX (HD-MTX). A transient increase in ALT levels with no clinical significance is common after HD-MTX in patients with childhood ALL [12], and ALT levels should return to normal levels within two weeks [13]. Three patients’ highest ALT levels were observed at 916 or 917 days after diagnosis (Table 1), which is more than two and a half years (912 days). For those patients, it was confirmed from the medical records that maintenance therapy was, unusually, still ongoing but coming to an end, and the ALT levels were analyzed as a part of the investigations at the end of treatment. Aspartate aminotransferase, alkaline phosphatase, and gamma–glutamyl transferase levels were not available for all patients because the levels are not routinely followed during treatment and therefore are not reported. The *TPMT* genotype was specified in the patient’s medical records. MeMP levels were determined from the ALL2008 maintenance metabolite study [14], and they are not routinely analyzed during therapy. Data for severe adverse events (SAE) were collected from the NOPHO database [15].

**Table 1:** POLG1 variant, the highest serum alanine aminotransferase and bilirubin values during maintenance therapy with the CTCAE v5.0 grading, and methylated 6-mercaptopurine metabolites in the 34 patients with acute lymphoblastic leukemia.

Patient	POLG1 variant	ALT (U/l)	CTCAE grade (ALT)	Days after dg for highest ALT value	Bil (µmol/l)	CTCAE grade (Bil)	Days after dg for highest Bil value	MeMP (highest, nmol/mmol Hgb)	MeMP (lowest, nmol/mmol Hgb)	MeMP (mean, nmol/mmol Hgb)	Treatment risk group	SAE
1	p.Q1236H	295	3	899	38	2	184	<60	<60	<60	IR	Bleeding, pancreatitis
2	p.A467T	257	3	896	10	0	896	NA	NA	NA	IR	
3		298	3	897	21	0	190	33072	1760	14952	SR	
4		292	3	916	12	0	916	30318	<60	6716	SR	Paralysis
5	p.Q1236H	268	3	258	14	0	433	16135	<60	4638	HR	
6	p.Q1236H	304	3	907	16	0	907	31299	<60	10583	IR	Osteonecrosis, paralysis
7		631	3	893	11	0	741	9418	<60	1748	SR	Paralysis
8		58	1	236	12	0	907	14334	426	5759	IR	Intensive care, somnolence
9		180	2	917	21	0	917	40790	<60	12144	SR	
10		294	3	917	14	0	841	18507	<60	5572	SR	Osteonecrosis, paralysis
11		334	3	875	18	0	875	24313	<60	7712	IR	Osteonecrosis
12		545	3	673	6	0	882	42866	722	9729	HR	
13		180	2	896	10	0	476	<60	<60	<60	IR	Anaphylaxis, intensive care, paralysis
14	p.E1143G	148	2	193	22	0	900	25224	<60	8038	SR	
15		168	2	728	25	0	728	56293	<60	11184	IR	Anaphylaxis
16		74	1	586	10	0	907	13621	783	7045	SR	
17	p.Q1236H	209	3	875	15	0	533	21238	<60	6108	IR	Paralysis
18	p.Q1236H	1145	4	659	341	4	550	21836	<60	4926	SR	Osteonecrosis, paralysis
19		61	1	245	8	0	882	NA	NA	NA	IR	Anaphylaxis, paralysis
20		230	3	561	12	0	903	12118	<60	2542	IR	
21		58	1	900	9	0	900	1191	<60	476	IR	
22		538	3	142	10	0	631	10988	<60	4828	SR	Intensive care, paralysis, PRES, somnolence
23		490	3	319	23	0	899	19825	13500	16372	SR	Pancreatitis, paralysis
24		621	3	766	17	0	794	7665	<60	1881	IR	
25		328	3	638	9	0	661	13389	851	6314	HR	Fungal infection, osteonecrosis
26	p.Q1236H	615	3	616	35	1	609	9072	1144	4350	SR	
27		303	3	483	26	1	631	32901	1688	14546	SR	Osteonecrosis
28	p.Q1236H	214	3	908	17	0	900	8544	453	5316	IR	Anaphylaxis, fungal infection
29		856	4	902	15	0	470	25125	<60	6983	SR	
30	p.Q1236H	535	3	901	22	0	892	30658	<60	9843	IR	
31	p.G517V	2094	4	595	17	0	596	4514	<60	2230	SR	
32		276	3	897	22	0	119	10780	<60	2977	SR	Osteonecrosis, paralysis
33		64	1	232	NA	NA	NA	18227	<60	1796	IR	Intensive care
34	p.E1143G	103	1	363	46	2	369	25193	<60	9413	SR	Osteonecrosis, paralysis

*ALT* serum alanine aminotransferase, *Bil* bilirubin, *CTCAE* common terminology criteria for adverse events, *dg* diagnosis, *HR* high-risk treatment group, *IR* intermediate risk treatment group, *MeMP* methylated 6-mercaptopurine metabolites, *NA* not available, *PRES* posterior reversible encephalopathy syndrome, *SAE* severe adverse event, *SR* standard risk treatment group.

This study was approved by the Regional Ethics Committee of the Northern Ostrobothnia Hospital District, Finland, and it was conducted in accordance with the Declaration of Helsinki. Informed consent was obtained from the patients or their legal guardians before their participation in the study.

### DNA extraction

DNA was extracted, depending on its availability for each patient, from diagnostic bone marrow samples (for 8 patients), from both diagnostic bone marrow and remission blood samples (for one patient), and from either blood or bone marrow samples at remission (for 25 patients; Supplementary Table S1), using the Gentra Puregene Blood Kit (Qiagen, Hilden, Germany) according to the manufacturer’s instructions.

### PCR and restriction fragment length polymorphism

The DNA samples were amplified by PCR to screen the nine common *POLG* variants, namely, c.752C>T, c.1399G>A, c.1402A>G, c.1550G>T, c.1760C>T, c.2165G>A, c.2243G>C, c.3428A>G, and c.3708G>T (p.T251I, p.A467T, p.N468D, p.G517V, p.P587L, p.R722H, p.W748S, p.E1143G, and p.Q1236H, respectively). PCR was performed using AmpliTaq Gold® DNA Polymerase (Applied Biosystems, Foster City, CA, USA) and Biotools DNA Polymerase (5U/µl; Biotools B&M Labs S.A., Madrid, Spain). Restriction fragment length polymorphism (RFLP) was performed using FastDigest (Thermo Fisher Scientific, Waltham, MA, USA) and NEB enzymes (New England Biolabs, Ipswich, MA, USA) and following the manufacturers’ instructions. For the p.W748S variant, DNA samples were amplified by allele-specific PCR with locked nucleic acid (LNA) primers (Exiqon A/S, Vedbaek, Denmark, and Sigma-Aldrich, St.Louis, MO, USA). PCR products were visualized using 1.5% agarose gel (Standard Agarose—Type LE, BioNordika, Helsinki, Finland) and a Safe Imager™ Blue-Light Transilluminator (Invitrogen, Carlsbad, CA, USA). The digested RFLP products were visualized using 1.5% agarose gel (Standard Agarose—Type LE, BioNordika, Helsinki, Finland), 2–3% MetaPhor gel (Lonza Rockland Inc., Rockland, ME, USA), or 3% 3:1 HRB™ agarose gel (Amresco Inc., Solon, OH, USA), depending on the fragment size.

### Expand long-template PCR and sequencing

The DNA samples were also amplified using long PCR (expand long-template PCR) to screen possible mtDNA deletions [16]. Whole mtDNA amplification was carried out using Phusion High-Fidelity DNA Polymerase (2 U/µl; Thermo Fisher Scientific), and PCR products were electrophoresed using 0.7% agarose gel (Standard Agarose—Type LE, BioNordika, Helsinki, Finland) at 70 V for 4.5 h. Sanger sequencing [17] was performed to confirm the presence of the p.A467T/p.N468D variant detected by RFLP in patient 2 (Table 1) and to cover the 23 coding exons and the exon–intron boundaries of the *POLG* gene in two patients showing signs of clinical liver dysfunction (patient 18 and patient 31; Table 1). PCR products were purified using exonuclease I and shrimp alkaline phosphatase [18], and sequencing analysis was run using an Applied Biosystems 3500xL Genetic Analyzer (Biocenter Oulu, Finland) with a Big-Dye Terminator v1.1 Cycle Sequencing Kit (Applied Biosystems). The *POLG* sequences were aligned with a reference sequence NG_008218.1 (available in the National Center for Biotechnology Information [NCBI] database) using the Sequencher 5.0 sequence analysis software demo (Gene Codes Corporation, Ann Arbor, MI, USA).

## Results

### Patients with severe hepatotoxicity during maintenance therapy

Three patients (3/34; 9%) showed grade 4 ALT levels (20 times above the upper normal limit) based on the Common Terminology Criteria for Adverse Events v5.0 (CTCAE) [19] with peaks at 856, 1145, and 2094 (U/l) (Table 1). No other SAE appeared to associate with the POLG1 variants (Table 1).

#### Patient with severe hepatotoxicity during maintenance therapy without elevated MeMP levels

A four-year-old female diagnosed with precursor B-cell ALL with a somatic t(12;21) translocation developed symptomatic hepatotoxicity with jaundice and increased central and peripheral periportal echogenicity as evidenced by liver ultrasound (US) findings and had a grade 4 ALT value. She harbored a heterozygous *POLG* c.1550G>T (p.G517V) variant (background population frequency: 0.0047; https://gnomad.broadinstitute.org/) [20]. She did not have excessively elevated MeMP levels (median during the episode: 2230 nmol/mmol Hgb), and she had a wild-type *TPMT* genotype. The patient was otherwise healthy. She was treated according to the NOPHO-ALL2008 SR protocol, and she is currently in remission.

**Fig. 1:**
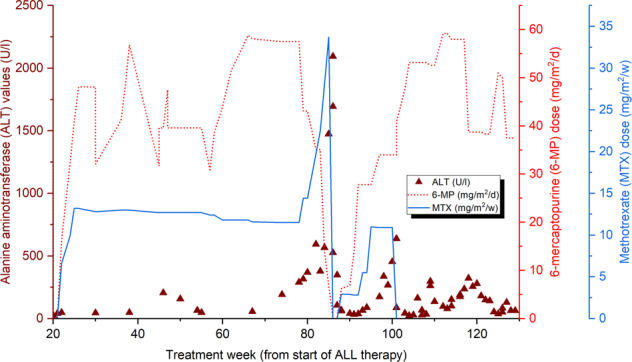
Graph of the serum alanine aminotransferase (ALT) levels and 6-mercaptopurine (6-MP) and methotrexate (MTX) doses during maintenance therapy of patient with the POLG1 p.G517V variant. Hepatotoxicity occurred at treatment week 82, and the serum ALT levels peaked at 2094 U/l at week 86. MTX dose was increased from 14 mg/m^2^/w to 34 mg/m^2^/w before first cessation. At the same time 6-MP dose was gradually reduced from 43 mg/m^2^/d to 7 mg/m^2^/d, and then ceased. Both medications were recommenced at week 86, and the 6-MP dose was gradually increased to the pre-episode level (59 mg/m^2^/d) but MTX therapy was stopped completely because ALT levels started to rise again.

She developed hepatotoxicity at treatment week 82 during maintenance therapy after an increase in MTX and 6-MP doses to achieve the target leukocyte count (Fig. 1; Supplementary Table S2). Maintenance therapy was initiated at week 20 according to the SR protocol and consisted of daily doses of 6-MP (starting dose: 75 mg/m^2^) and weekly doses of MTX (starting dose: 20 mg/m^2^), which are set by the target leukocyte count and drug tolerance [1]. The patient reported nausea before elevation was detected in serum ALT levels, which peaked at 2094 U/l. The MeMP levels of the patient remained within the reference range (<20 000 nmol/mmol Hgb).

**Fig. 2:**
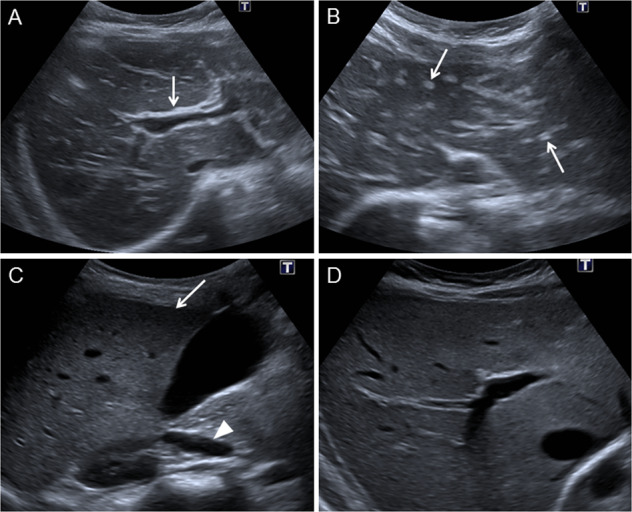
Liver ultrasound (US) images showing hepatic findings of a patient with the POLG1 p.G517V variant. Liver US performed at week 86 during maintenance therapy showed increased periportal echogenicity in the main portal vein (**A**, arrow) and segmental portal branches (**B**, arrows). Liver US performed at week 90 demonstrated regression of periportal echogenicity (**C**), but it showed mild hyperechogenicity of liver parenchyma (arrow) compared with that of the kidney (arrowhead). Follow-up US one year after the first US still showed slight hyperechogenicity of liver parenchyma (**D**).

Liver US was performed at week 86, showing increased central and peripheral periportal echogenicity (Fig. 2A, B). A liver biopsy was not performed. Immunoglobulins, smooth muscle and antinuclear antibodies, and viral tests, including cytomegalovirus, Epstein–Barr virus, and hepatitis viruses, were all analyzed as normal. All medications, including sulfadiazine/trimethoprim, were discontinued. The patient’s ALT levels decreased after the cessation of chemotherapy. Liver US performed in week 90 showed a decrease in the periportal echogenicity but an increase in the parenchymal echogenicity of the liver compared with that of the kidneys, suggesting hepatotoxic medication-induced hepatic steatosis (Fig. 2C).

After normalization of the patient’s ALT levels, 6-MP and MTX treatments were reinitiated at week 88, with a decreased dose. However, the ALT levels elevated again, and the increase in MTX administration appeared to have a more negative effect than that of 6-MP, so MTX therapy was ended completely at week 101 (Fig. 1; Supplementary Table S2). In the same week, prednisolone and vincristine were added to the maintenance therapy to enable the dose reduction of 6-MP and to compensate for the cessation of MTX. The upper limit of the target leukocyte count for maintenance therapy was increased from 3.0 to 3.5 (E9/l). With these measurements, ALT levels could be maintained within an acceptable range. Prednisolone and vincristine therapy ceased at week 108. Pentamidine isethionate was substituted for sulfadiazine/trimethoprim until week 110. Mild hyperechogenicity of the liver parenchyma was observed one year after the first US (Fig. 2D).

Low doses of oral MTX combined with 6-MP during maintenance appeared intolerable for the patient. During the HD-MTX treatment, hepatotoxicity was not detected, and the liver enzymes remained within an acceptable range. The HD-MTX treatment resulted in mucositis, and because of that, the administration of HD-MTX was postponed once during induction.

#### Patients with severe hepatotoxicity during maintenance therapy with elevated MeMP levels

The remaining two patients with grade 4 ALT levels had high MeMP levels (>20 000 nmol/mmol Hgb), and both were *TPMT* wild type. One of them had a solitary high ALT value (856 U/l) at the end of treatment (day 902) and was asymptomatic, having previous ALT levels mainly below 200 U/l. This patient harbored none of the screened *POLG* variants.

The second patient had a *POLG* c.3708G>T (p.Q1236H) variant with a peak ALT level of 1145 U/l, which did not influence the maintenance drug doses and was not associated with liver failure. After two weeks, the patient’s ALT levels had dropped to 350 U/l. In the earlier phase of maintenance therapy, the patient developed hypoglycemia and nausea, with subsequent hyperbilirubinemia, jaundice, and itch. These symptoms are consistent with common side effects associated with 6-MP treatment and elevated MeMP levels [4]. During the episode, the US revealed cholecystitis (irritated gall bladder) but no dilated bile ducts and normal liver parenchyma. Bilirubin rose to 341 µmol/l (CTCAE grade 4; Table 1), but the ALT level was low (14–155 U/l), alkaline phosphatase was normal (276 U/l), and gamma–glutamyl transferase was CTCAE grade 1 (100 U/l). Platelets decreased to 66 E9/l and hemoglobin to 103 g/l, but total white blood cell (2.5 E9/l at the lowest) and neutrophil (1.45 E9/l) counts were within the target range. 6-MP was ceased for 4 weeks, and MTX was increased concurrently.

### *POLG* variants

Of the nine common POLG1 variants screened in this study, four different variants, all heterozygous, were detected in 12 of the 34 patients (Table 1). Eight patients had only diagnostic bone marrow samples available, and of them, four patients had a POLG1 variant. The p.G517V variant was found only in the patient with clinically significant hepatotoxicity. To rule out a somatic variant, the p.G517V variant was detected in both diagnostic bone marrow and blood at follow-up. In addition to one patient with grade 4 ALT and bilirubin levels, seven patients harbored the p.Q1236H variant and had grade 3 ALT levels but showed no signs of clinical liver dysfunction. Of those, three had high MeMP levels. In addition, one patient harbored the p.A467T variant with grade 3 ALT with no available MeMP data. Two patients harbored the p.E1143G variant with normal or low ALT levels. In total, 21 of the 34 (62%) study patients had a grade 3 ALT level.

### Mitochondrial DNA deletions

None of the study patients showed mtDNA deletions, indicating that the chemotherapy did not affect the integrity of mtDNA in blood and bone marrow cells. However, mtDNA deletions were not analyzed in the patients’ hepatocytes.

## Discussion

We identified a patient who developed severe hepatotoxicity during ALL maintenance therapy with a wild-type *TPMT* allele and without concomitant elevated MeMP levels. She harbored a heterozygous *POLG* c.1550G>T (p.G517V) variant, which was not detected in the other patients.

The p.G517V variant locates in the spacer region of POLG1, which lies between the exonuclease and polymerase domains [5]. The spacer region is involved in multiple functions, including protein–protein interactions, enzyme activity, and DNA-binding affinity, and homozygous or compound heterozygous variants in the spacer region are disease associated [21, 22]. POLG1-related diseases present variable clinical manifestations, from infantile-onset epilepsy and liver failure to late-onset myopathy and ataxia [5]. The p.G517V variant has been associated with hepatocerebral syndromes, myopathy, peripheral neuropathy, and progressive external ophthalmoplegia syndrome [5, 23]. However, it is debated whether the p.G517V variant is a frequent single nucleotide polymorphism or a disease-associated pathogenic variant [5, 23]. Biochemical analysis has indicated that recombinant human POLG1 with the p.G517V variant retains 80–90% of its wild-type activity [24].

POLG1 variants cause variable phenotypes, and one considered mechanism is that the variability arises from ecogenetic variants (i.e., silent variants) that, under certain conditions, such as medication or epigenetic changes, present the clinical phenotype of POLG1 dysfunction [25]. This genetic modifier role, where different *POLG* alleles induce risk for clinical phenotypes, is present in VPA-induced mitochondrial liver failure [25]. The p.G571V variant may act as a genetic modifier that introduces mitochondrial toxicity and impairs Pol γ activity under specific medication or in combination with other gene variants. Staropoli *et al*. describe a patient with *CLN5*-associated neuronal ceroid lipofuscinosis harboring an additional heterozygous POLG1 p.G517V variant with decreased mtDNA copy number resulting in an atypical clinical phenotype [26]. Our hypothesis is that the genetic modifier role of the p.G517V variant may explain the hepatotoxicity triggered by maintenance drugs MTX and 6-MP in our study patient.

Our patient with the p.G517V variant seemed not to tolerate low-dose oral MTX, as an increase in the MTX dose with a concomitant decrease in the 6-MP dose resulted in clinical hepatotoxicity with elevated transaminases. The liver is a highly energy-dependent organ and, as such, is susceptible to drug-induced mitochondrial dysfunction, which is common in drug-induced liver failure [7, 27]. MTX has been shown to impair mitochondrial respiratory chain function [7]. In addition, oxidative stress caused by increased reactive oxygen species (ROS) and reduced glutathione levels may be a mechanism for MTX-induced mitochondrial toxicity [28–30]. MTX-induced impairment of the mitochondrial respiratory chain leads to reduced fatty acid oxidation and increased fatty acid accumulation (i.e., macrovacuolar steatosis), thus resulting in drug-induced hepatic steatosis [7]. This is in line with our patient, whose US revealed hepatic steatosis. Non-alcoholic steatohepatitis (NASH) has been observed in liver biopsies of patients with rheumatoid arthritis receiving low doses of oral MTX, although those patients had additional risk factors for NASH [31]. A recent study showed that therapeutic levels of low-dose MTX resulted in the accumulation of ROS and impairment of the mitochondrial respiratory chain on human hepatoma and hepatic stellate cells [30]. VPA-induced liver failure associated with POLG1 variants usually requires 2–3 months of VPA administration, ranging from 4 to 26 weeks, before the onset of symptoms [25, 32, 33]. Our study patient showed signs of hepatotoxicity after 62 weeks of oral antimetabolite therapy, and the symptoms occurred rapidly after increasing the MTX dose. The HD-MTX treatment did not cause clinically noticeable liver dysfunction for our patient. We hypothesize that the p.G517V variant imposes a risk for hepatotoxicity with long-term administration of low-dose oral MTX in connection with 6-MP during maintenance. However, this needs to be validated in further studies.

Pol γ has been shown to be affected by drugs used to treat ALL, especially thiopurines; chemotherapeutic drugs such as 6-thioguanine (6-TG) are incorporated into mtDNA, where they are oxidized [34]. These oxidized forms of DNA-incorporated 6-TG effectively inhibit Pol γ, thus decreasing mitochondrial replication, transcription, and protein synthesis [34]. Impaired protein synthesis and respiratory chain function cause depletion of adenosine triphosphate for energy usage [35]. Gene variants disrupting mitochondrial function may be risk factors for antimetabolite (6-MP, 6-TG, and MTX) therapy-induced hepatotoxicity in ALL. In previous genome-wide association or single-nucleotide polymorphism studies, *POLG* has not been associated with hepatotoxicity [36–38]. Liu et al. found that the *PNPLA3* I148M variant is associated with elevated transaminases in pediatric ALL patients, and the gene has been linked to ALT elevations in other populations [36]. Known factors for 6-MP-related toxicity and hepatotoxicity are *TPMT* and *NUDT15* variants [39, 40]. *NUDT15* variants are more common in Asian populations [39]. In addition, polymorphisms of *ITPA* and *ABCB1* have been associated with 6-MP-related hepatoxicity [41, 42]. As part of the NOPHO-ALL2008 treatment protocol, only *TPMT* genotyping was performed [40]. In this study, only *POLG* variants were analyzed, in addition to routine genotyping of *TPMT*, excluding other genetic variants that may cause drug-induced hepatotoxicity.

Aside from one patient with p.G517V and clinically severe hepatotoxicity, no significant correlation between POLG1 variants and ALT or bilirubin levels was observed in the patient cohort. One patient with the p.Q1236H variant developed clinical liver failure but conversely had high MeMP levels and hyperbilirubinemia with low ALT levels during the episode. The patient also had hypoglycemia during the episode, which has been associated with POLG1-related liver failure in Alpers patients with impaired mitochondrial respiratory chain activity [25], but it is also a common adverse effect of 6-MP treatment and high MeMP levels [4]. The p.Q1236H variant is common in Northern European populations, with a 14.8–15.9% prevalence in the Finnish population [21, 43]. It is considered a polymorphism, but it may have a role as a genetic modifier [21, 25]. Recently, the p.Q1236H variant has been shown not to associate with VPA-induced liver toxicity [33], in contrast to previously published studies [44].

## Conclusion

This research may be considered a pilot study for the role of POLG1 variants in ALL maintenance therapy. The p.G517V variant was detected in the patient who developed clinically severe hepatotoxicity during oral maintenance therapy with MTX and 6-MP, which was not associated with 6-MP metabolism, as indicated by the presence of a wild-type *TPMT* variant and normal MeMP levels. The p.G517V variant was not detected in other cohort patients, but a limitation of this study was its small patient cohort. These findings call for larger studies of the impact of POLG1 variants on hepatotoxicity during maintenance therapy, including interactions with other variants influencing 6-MP and MTX drug disposition and tolerance.

## Supplementary information

Supplementary table S1

Supplementary table S2

## Data Availability

The datasets generated and/or analyzed during the current study are available from the corresponding author upon reasonable request.
